# Suprapubic tube versus urethral catheter drainage after robot-assisted radical prostatectomy: a systematic review and meta-analysis

**DOI:** 10.1186/s12894-017-0312-5

**Published:** 2018-01-05

**Authors:** Zhongyu Jian, Shijian Feng, Yuntian Chen, Xin Wei, Deyi Luo, Hong Li, Kunjie Wang

**Affiliations:** Department of Urology, Institute of Urology (Laboratory of Reconstructive Urology), West China Hospital, Sichuan University, No. 37 Guo Xue Xiang, Chengdu, Sichuan 610041 People’s Republic of China

**Keywords:** Prostate cancer, Prostatectomy, Robotics, Suprapubic catheter, Urethral catheterization

## Abstract

**Background:**

Prostate cancer is one of the most common cancers in the elderly population. The standard treatment is radical prostatectomy (RARP). However, urologists do not have consents on the postoperative urine drainage management (suprapubic tube (ST)/ urethral catheter (UC)). Thus, we try to compare ST drainage to UC drainage after robot-assisted radical prostatectomy regarding to comfort, recovery rate and continence using the method of meta-analysis.

**Methods:**

A systematic search was performed in Dec. 2017 on PubMed, Medline, Embase and Cochrane Library databases. The authors independently reviewed the records to identify studies comparing ST with UC of patients underwent RARP. Meta-analysis was performed using the extracted data from the selected studies.

**Results:**

Seven studies, including 3 RCTs, with a total of 946 patients met the inclusion criteria and were included in our meta-analysis. Though there was no significant difference between the ST group and the UC group on postoperative pain (RR1.73, P 0.20), our study showed a significant improvement on bother or discomfort, defined as trouble in hygiene and sleep, caused by catheter when compared two groups at postoperative day (POD) 7 in ST group (RR2.05, P 0.006). There was no significant difference between the ST group and UC group on urinary continence (RR0.98, P 0.74) and emergency department visit (RR0.61, P 0.11). The rates of bladder neck contracture and other complications were very low in both groups.

**Conclusion:**

Compared to UC, ST showed a weak advantage. So it might be a good choice to choose ST over RARP.

## Background

Prostate cancer is one of the most common cancers in the elderly population. In fact, prostate cancer is the most common cancer in male. It was estimated that in 2014, 233,000 men were diagnosed with prostate cancer and 29,480 men died of this disease [1]. Radical prostatectomy (RP) is an effectively therapy for those who are clinically diagnosed with localized prostate cancer [2]. Urethral catheter (UC) is traditionally used in RP, not only for drainage of the bladder but also protecting the anastomosis and promoting the healing process.

Compared to the retropubic approach, robot-assisted radical prostatectomy (RARP) had a lower incidence rate of anastomotic stricture [3]. Some studies reported uneventful early catheter removal after RARP [4, 5]. Therefore, the use of UC might not be as crucial as previously envisaged. On the other hand, complaints about the discomfort associated with UC were commonly seen in the clinic. In order to improve the life quality of patients, some researchers are exploring whether replacing UC with percutaneous suprapubic tube (ST) after RARP is a better choice [6–12].

The first report of catheter-less technique after RARP was published in 2008, which showed favorable results in terms of postoperative pain and early recovery of continence [6]. Later researches reported conflicting results in postoperative pain after surgery [7, 8]. Until now, there was no consents or systematic review focusing on ST and UC after RARP. We searched and analyzed the data from the literatures to compare postoperative pain, urinary continence and other related outcomes between ST and UC after RARP surgery.

## Methods

### Search strategy

A systematic search was performed in Dec. 2017 on PubMed, Medline, Embase and Cochrane Library databases. The following MeSH terms and their combinations were searched in [Title/Abstract]: suprapubic, catheter, catheterization, tube, robotic, radical, prostatectomy, prostate cancer.

### Inclusion and exclusion criteria

The inclusion criteria were studies comparing UC and ST for RARP, including randomized controlled trials (RCT), case-control and cohort studies. Our study was limited to human subjects, gender (male), and languages (English and Chinese). Conference abstracts, case reports, letters or reviews were excluded from further analysis.

### Data extraction

Two authors reviewed the titles, abstracts and full texts of included studies independently. If disagreement appeared, a senior author was asked to make the final decision. Data was extracted from the included eligible studies. If data was presented as pictures rather than numbers, GetData Graph Digitizer (version 2.26) was used to extract relevant data. The information extracted from the study are listed below: postoperative pain, bother or discomfort by catheter, urinary continence, bladder neck contracture (BNC), emergency department visit and complications.

### Quality assessment and statistical analysis

The quality of RCTs was assessed using the Cochrane risk of bias tool. The quality of case-control studies was assessed using the modified Newcastle-Ottawa scale (The total score is nine, studies score six or above were considered as high quality). Data analysis was performed with Review Manager (RevMan 5.3, Cochrane Collaboration, Oxford, UK).

The risk ratio (RR) and weighted mean difference (WMD) were used to compare dichotomous and continuous variables, respectively. And the 95% confidence intervals of the statistics were presented. Heterogeneity was tested using the chi-square test. A random effects model was utilized if *I*^2^ > 50%, otherwise the fixed-effects model was used. *P* < 0.05 was defined as statistically significant different.

## Results

### Description of included studies and quality assessment

**Fig. 1:**
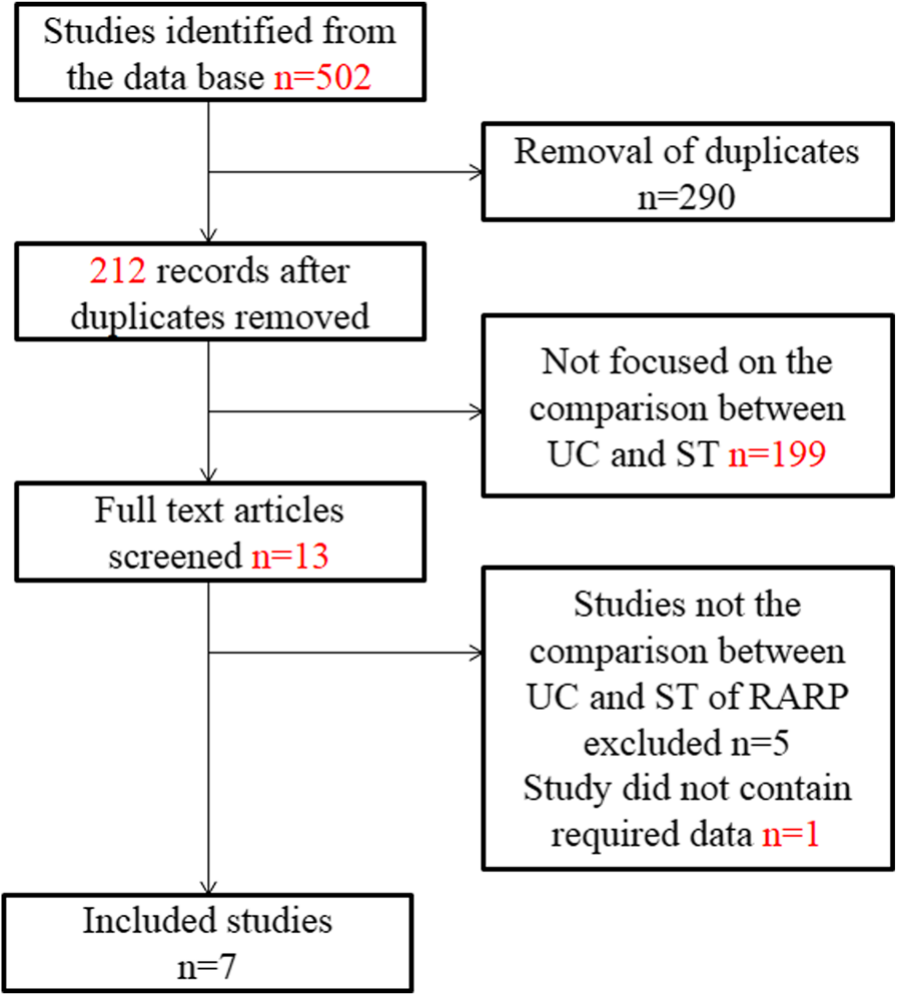
Literature analysis and data acquisition; UC=Urethral Catheter, ST = Suprapubic Tube

A total of 502 articles were acquired through literature search and screened for eligibility. 212 articles were identified after removal of duplicates in the four database mentioned above. 199 articles were excluded because they were not focused on the comparison between UC and ST of RP after screening the titles and abstracts. Three studies were excluded because they were comments. One descriptive study only focused on ST, and another study about retropubic radical prostatectomy (RRP) were also excluded. We also excluded one study for it did not contain required data in numeric format, but present using figure, which did not show the standard deviation. Finally, seven studies with 946 patients were included in this systematic review (Fig. 1).

The characteristics of the included studies were shown in Table 1. Among these studies, three were RCTs reaching the level of evidence 1b [8, 11, 12]. One cohort, non- randomized study reached the level of evidence 2b [6]. Two case-control studies compared contemporary series of patients reached the level of evidence 3b [9, 10]; and one case-control study using historical series as controls reached the level of evidence 4 [7].

**Table 1:** Characteristics of included studies

Study	LOE	Design	N		follow up	Time for removal of UC and ST		Pain	Continence definition	Quality
			UC	ST		UC	ST			
Tewari A 2008 [6]	2b	pro	20	10	6 months	POD 7	POD 7	questionnaire	0-1pad/day	8
Krane LS 2009 [7]	4	Retro	50	202	6-12 months	POD 7	POD 7	FPS-R scale (0–10)	0-1pad/day	6
Prasad SM 2014 [8]	1b	pro-random	29	29	N	POD 7	POD 7 (UC removed POD1)	VAS scale (0–10)	N	RCT
Afzal MZ 2015 [9]	3b	Retro	174	51	N	POD 8	POD 6–9 (UC removed POD1)	N	0-1pad/day	7
Morgan MS 2016 [10]	3b	Retro	65	94	>3 months	POD 7–10	POD 9–10 (UC removed POD1)	questionnaire	N	7
Martinschek A 2016 [11]	1b	pro-random	35	27	1 year	N	N	VAS scale (0–10)	N	RCT
Harke N [12]	1b	pro-random	80	80	2 years	POD 5	N (UC removed POD1)	NRS (0–10)	0-1pad/day	RCT

*ST* Suprapubic tube, *UC* urethral catheter; *Pro* prospective, *Random* randomised, *Retro* retrospective, *LoE* level of evidence, *POD* postorerative day, *N* not gived, *VAS* visual analog scale, *NRS* numeric rating scale, *FPS-R scale* Faces Pain Score-Revised, *RCT* randomized controlled trail

### Postoperative pain

**Fig. 2:**
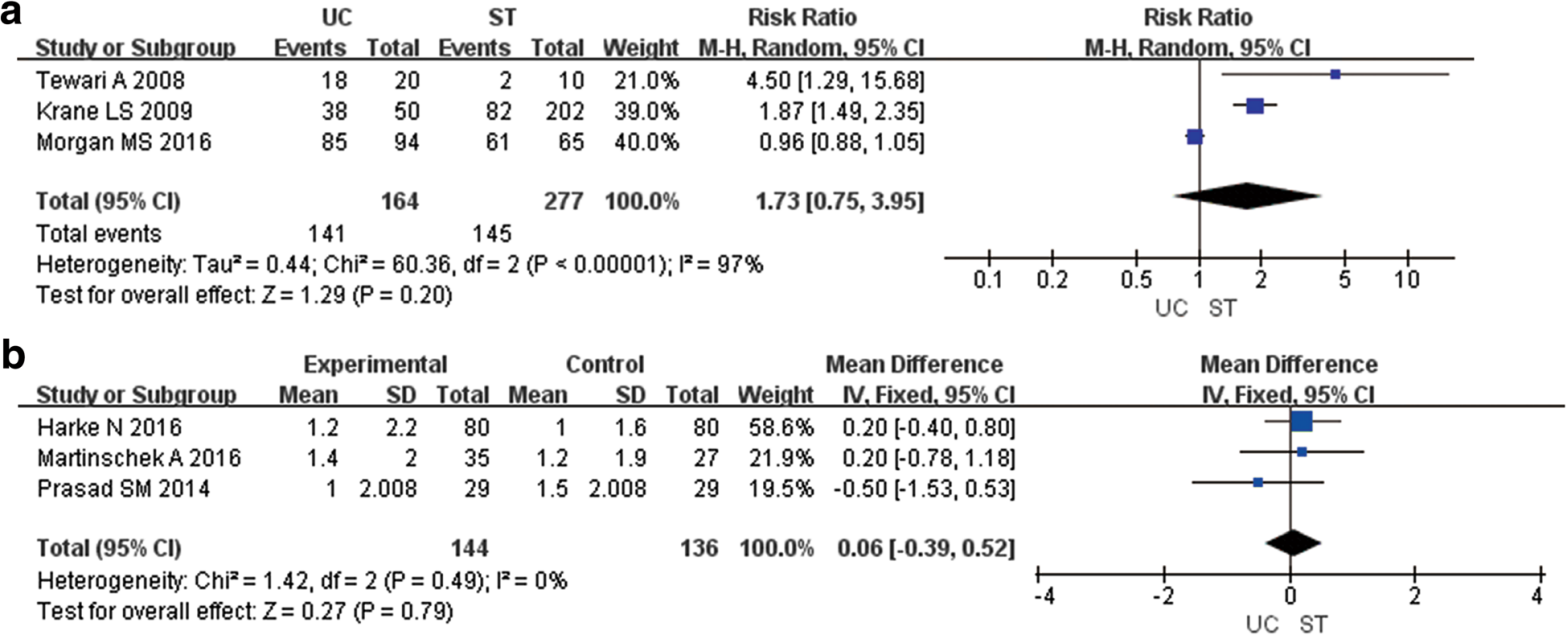
**a** Forest plot of RR for Postoperative pain at POD 6–7; UC=Urethral Catheter, ST = Suprapubic Tube, RR = Risk Ratio, POD = Postoperative Day. **b** Forest plot of WMD for Postoperative pain at POD 1; UC=Urethral Catheter, ST = Suprapubic Tube, WMD = Weighted Mean Difference, POD = Postoperative Day

A total of six studies reported postoperative pain at postoperative Day (POD) 6 [7, 11, 12] or POD 7 [6, 8, 10]. We divided the patients into two groups (with pain and without pain). Three articles eligible for the meta-analysis (441 patients) were shown in the Fig. 2a [6, 7, 10]. There was no significant difference between UC and ST group (RR1.73; 95% CI 0.75, 3.95; P 0.20) (Fig. 2a).

Four studies reported postoperative pain at POD 1 [8, 11, 12] or POD 2. [7] Meta-analysis of the first three RCTs (280 patients) showed that ST group had not significantly decreased pain at POD 1 compared to the UC group (WMD 0.06; 95% CI -0.47, 0.59; P 0.79) (Fig. 2b). The fourth study [7] reported that patients in the ST group had significantly decreased pain at POD 2 compared to the UC group (*P* < 0.001).

### Bother or discomfort by catheter

**Fig. 3:**
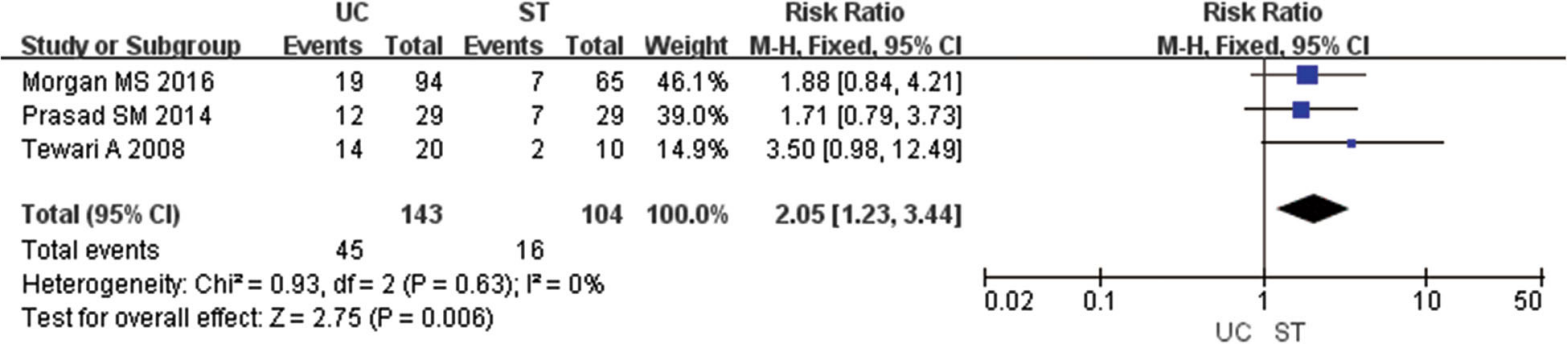
Forest plot of RR for bother or discomfort by catheter; UC=Urethral Catheter, ST = Suprapubic Tube, RR = Risk Ratio

Bother or discomfort was defined as the trouble from the hygiene and sleep. The meta-analysis of three studies (247 patients) [6, 9, 10] showed a statistically significant advantage on the rate of bother or discomfort in favour of the ST compared to UC at POD 7 (RR2.05; 95% CI 1.23,3.44; P 0.006) (Fig. 3).

### Urinary incontinence

**Fig. 4:**
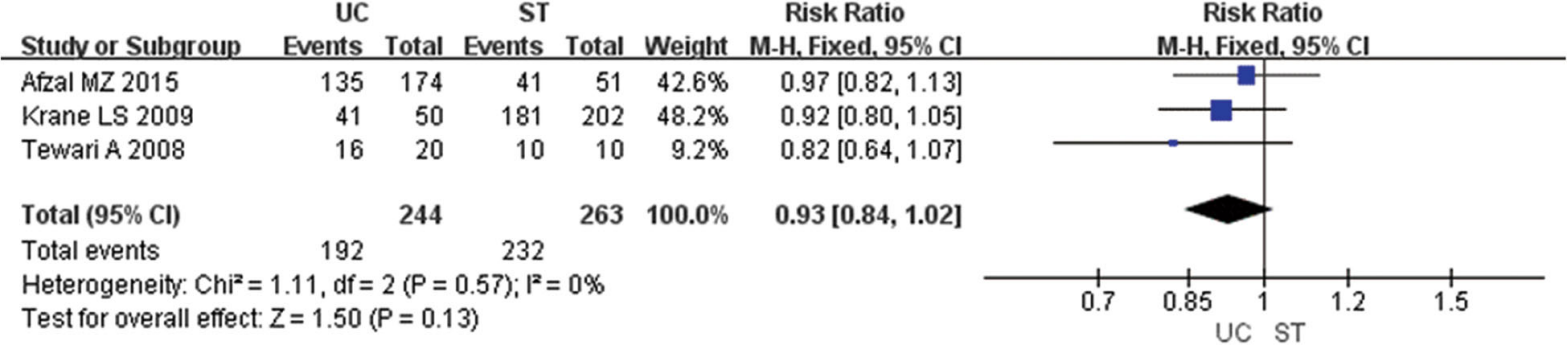
Forest plot of RR for urinary continence at 6 weeks after surgery; UC=Urethral Catheter, ST = Suprapubic Tube, RR = Risk Ratio

A total of 507 patients in three studies were included in the meta-analysis for urinary continence [6, 7, 9] (Fig. 4). Results of urinary continence showed no difference between UC group and ST group at 6 weeks after the surgery (RR0.93; 95% CI 0.84, 1.02; *P* 0.13).

### Emergency department visit and complications

**Fig. 5:**
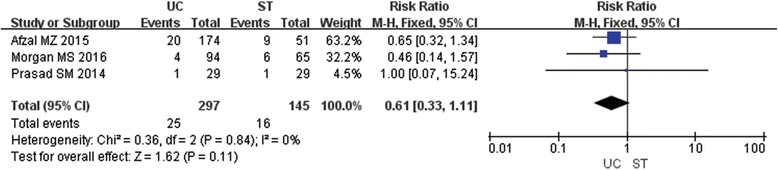
Forest plot of RR for emergency department visit; UC=Urethral Catheter, ST = Suprapubic Tube, RR = Risk Ratio

Three studies containing 442 patients in assessed the emergency department visit and complications in their studies. The result showed that there was no significant difference between the UC and ST group on the rate of emergency department visit (Fig. 5) (RR0.61; 95% CI 0.33,1.11; P 0.11) [8–10]. Two studies showed no significant difference on bladder spasms between the UC and ST group, 3/10 vs 8/20 (*P* > 0.05) [6] and 56/94 vs 40/65 (P 0.90) [10] respectively.

### Publication bias and sensitivity analysis

**Fig. 6:**
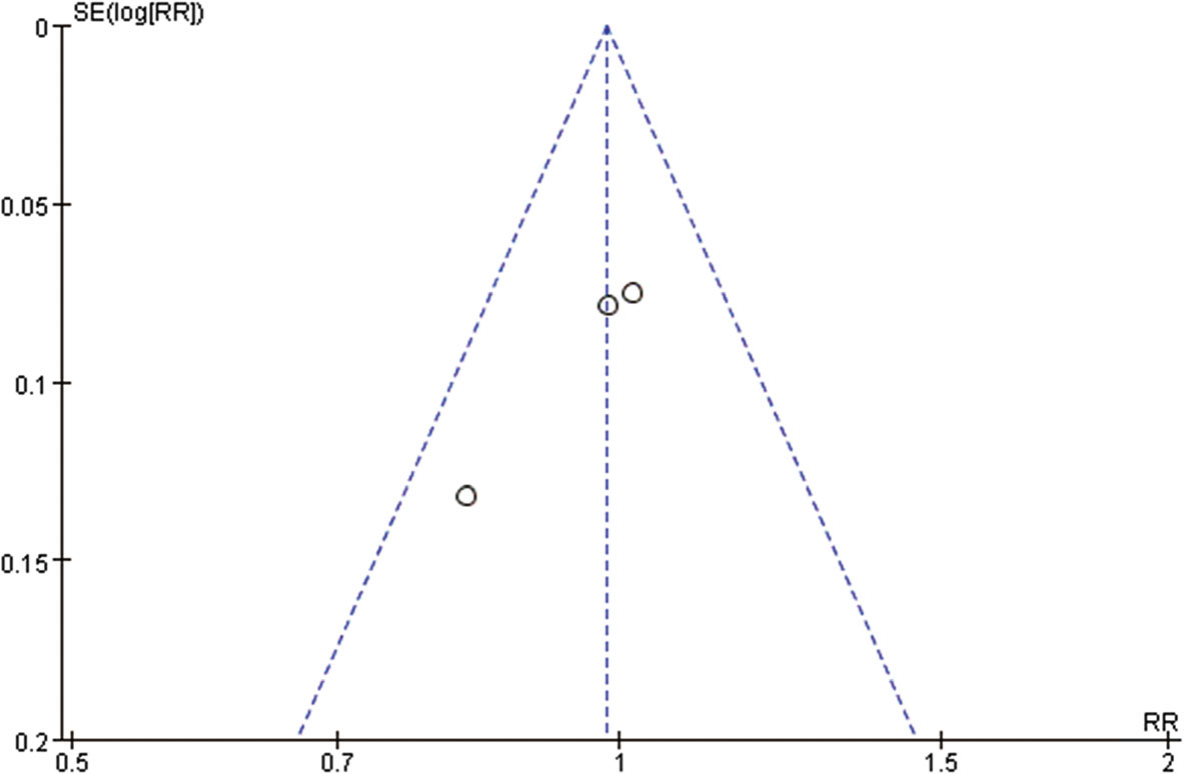
Funnel plot for urinary continence; RR = Risk Ratio

Figure 6 shows a funnel plot of the studies included in this meta-analysis that reported urinary continence. All studies were evenly distributed inside the 95% CIs, which indicated no obvious publication bias. The funnel plots of the studies reported pain, bother and emergency department visit showed the same results as the urinary continence.

Three RCT studies used visual analog scale (VAS) to evaluate the postoperative pain [8, 11, 12]. Meta-analysis of these three studies revealed no significant difference between the UC and ST group (WMD 0.06; 95% CI -0.39, 0.52; P 0.79) regarding the postoperative pain.

## Discussion

A recent systematic review including 42 trials indicated an advantage on suprapubic catheterization in terms of asymptomatic bacteriuria and pain compared to the urethral catheterization [13]. To our knowledge, UC was traditionally used in RP not only for the drainage of bladder but also for protecting the anastomosis and promoting healing. Lately, several studies have tried to use ST instead of UC after robot-assisted RARP to improve patient life quality. Outcomes of these studies were conflicting, and we systematically searched and collected the studies that compared UC and ST after RARP, and presented the first systematic review and meta-analysis on this topic.

The postoperative pain was a controversial topic [7, 8]. Our study demonstrated that there was no significant difference between the UC and ST group on postoperative pain at POD 7. The other three RCT studies showed similar result too [8, 11, 12]. Prasad et al. stated that the most severe discomfort of catheter was experienced in the evening on surgery day due to bladder spasms induced by the presence of foreign body. The next morning, discomfort from the catheter was eased considerably [14]. Postoperative pain at POD 1 also showed no significant difference between the two groups [8]. But when considering the penile pain, the UC group seemed to be more severe than the ST group according to Morgan et al. [10]. Another recently published study also demonstrated the postoperative pain is superior in ST group than in UC in POD 1 to 5. However, in POD 6, the difference were not statistically different in two groups anymore, which is consistent with our results [12]. So the postoperative pain maybe not associated with the kind of catheterization in the long term, but ST might have advantage in the short term.

Not surprisingly, our results showed a statistically significant advantage on the rate of bother or discomfort in favor of the ST group over the UC group at POD 7. As we all known, the catheterization will influence patients’ quality of life including sleep, generally hygiene and genital hygiene, in a bad way. Only one study evaluated the bother at POD 1 to 6, and the results were similar between two groups. Therefore, patients with UC were more bothersome than ST [11].

Regard to incontinence, Krane et al. assessed the urinary incontinence at 2 days, 7 days and 90 days [7]. 23 (46%) patients with UC and 101 (50%) patients with ST were continent at 2 days postoperatively. At 90 days, 41 (92%) of patients with UC and 181 (90%) of patients of ST were recovered from incontinence. But all of the above incontinence results showed no significant difference (*P* > 0.2 for all time points). Tewari also evaluated the percentage of patient urinary continence of UC and ST at 1 and 12 weeks, and the differences were not statistically significant, 20% vs 20% and 100% vs 98% respectively [6]. Another study showed a trend in favor of ST at five days after surgery (UC 3.1 ± 2.4 vs ST 1.6 2.6; P 0.0752) using urinary pads [11]. A longer follow-up study also found no difference between the two groups at twelve and twenty-four months [12]. These result cannot be combined using meta-analysis due to obvious heterogeneity. According to other previous researches, Sammon et al. found that patients using ST after RARP achieved earlier recovery of incontinence [15]. Moreover, a long-term follow-up study showed the recovery from urinary incontinence was prompted with. 68.7% of continence rate at 4 weeks and 82.6% at 8 weeks after surgery [16]. The rates of recovery from incontinence in the three studies included in our meta-analysis were all very high, 100%, 81% and 82% respectively [6, 7, 9]. But when compared to UC, ST showed no significant advantage in terms of recovery from incontinence at 6 weeks, which is similar to our meta-analysis results. It is still unclear about the mechanism of ST helps early continence, and this proposition need to be further examined with high quality evidence in the future.

In our included studies, six of them with 745 patients measured the incidence of bladder neck contracture at 6 months to 2 years after surgery [6–8, 11, 12]. BNC appeared in two patients (2/35) in the UC group, but none of the patient in the ST group (0/27) from the study of Martinschek et al. [11]. In the study of Harke et al., [12] urethral stricture appeared in one patient in each group. The patients in the rest of the studies and groups had no BNC. Among all included studies, only two patients (2/35) in the UC group had BNC, while no patient in ST group (0/37) had BNC [11]. Open and laparoscopic/ robotic surgeries suggested that early removal of urethral catheter (2 to 4 days following surgery) did not increase the rate of bladder neck contracture [16, 17]. Meanwhile, urethral stricture appeared in one patient in each group [12]. The patients in the rest of the studies and groups showed no BNC through with a follow-up ranged from 6 months to 1 year. Therefore, the safety of ST regarding to BNC was trustworthy.

In general, complication is important in the evaluation of the safety of a technique. Thus, emergency department visit and complications of both technique is relatively important. There was no significant difference between the UC and the ST group on the rate of emergency department visit in our study. Tewari et al. reported that none of the patients in both groups had retention requiring irrigation [6]. The study of Krane et al. showed that 10(5%) patients required urethral catheterization because of ST dislodgement (*n* = 5, 2.5%) or urinary retention (*n* = 5, 2.5%), and additionally three (6%) patients need recatheterization after removing urethral catheter due to urinary retention [7]. Afzal et al. found that eight patients with UC (5%) and 6 patients with suprapubic catheter (11%) had catheter-related problems after RARP (P 0.18), which are urinary retention after catheter removal, ST malfunction and clot retention [9]. In another study, complication rate was not significantly different between UC (4.3%) and ST (4.6%) group (P 0.9) [10]. Similarly, Urinary retention requiring catheterization after catheter removal happened once in each of the two groups and catheter blockage with resulting urinary retention occurred twice in each group [11]. Only one article mentioned the bacteriuria (defined as >10^5^ bacteria/ml of urine) which was found in 10.3% (UC) and 5.1% (ST) of the patients (P 0.35). Among them, two patient required antibiotic treatment [12]. Two studies’ results showed no significant difference on bladder spasms between the UC and the ST group [6, 10]. The rate of urinary retention was very low (<5%) in these studies [6, 7, 11]. These evidence suggested that ST and UC were both safe after RARP.

There were also some limitations in our study. First, the included RCTs had small sample sizes, and the level of evidence of other included studies was relatively low. Second, the surgeries were performed by different surgeons with varied surgical experience and skills. These differences might influence the result.

## Conclusion

Based on our results, it can be concluded that while there was no significant benefit on pain after surgery in patients with ST compared to UC after RARP, an obvious advantage was observed in favor of ST on bother and discomfort caused by the catheter, especially in short term (1–5 days). Patients with ST and those with UC reported a comparable high rate of continence recovery. Safety outcomes including BNC, emergency department visit and urinary retention were also not significantly different between the two methods. Thus, it may be a good choice to choose ST instead of UT in postoperative management of RARP patients.

## Abbreviations

BNC: Bladder neck contracture
POD: Postoperative day
RARP: Radical prostatectomy
RR: Risk ratio
RRP: Retropubic radical prostatectomy
ST: Suprapubic tube
UC: Urethral catheter
VAS: Visual analog scale
WMD: Weighted mean difference
